# Purchase Intention for Organic Food Products in Mexico: The Mediation of Consumer Desire

**DOI:** 10.3390/foods10020245

**Published:** 2021-01-26

**Authors:** Sandra N. Leyva-Hernández, Arcelia Toledo-López, Ana B. Hernández-Lara

**Affiliations:** 1 Instituto Politécnico Nacional, CIIDIR Oaxaca, Oaxaca 71230, Mexico; sleyvah1400@alumno.ipn.mx; 2 Department of Business Management, Universitat Rovira i Virgili, 43204 Reus, Spain; anabeatriz.hernandez@urv.cat

**Keywords:** organic food consumption, consumer attitude, consumer desire, green consumption, model of goal-directed behaviour, theory of planned behaviour

## Abstract

Socially responsible consumption benefits the environment, the consumer, and the producer. In Mexico, smallholder farmers are vulnerable, and the consumption of organic food products is low. Analysing the purchase intention of organic food products contributes towards generating the most appropriate marketing strategies. Previous models provide evidence that the attitude of the consumer is the biggest predictor of purchase intention. However, little is known about the results of the mediating effect of desire on said relationship. The objective of the study is to analyse the mediating effect of desire on the relationship between attitude and purchase intention. 204 consumers of organic food products were surveyed using a structured, self-administrated questionnaire or through face-to-face interviews, in established retail stores, alternative street markets, and via the web. It was found that when the benefits of organic food products to the consumer, environment, and smallholder farmers are evaluated favourably, then consumer desire is higher, and thus also purchase intention. Consumers have the highest purchase intention for organic food products when their desire to buy them to achieve a goal related with social, personal, and environmental benefits intervenes.

## 1. Introduction

Socially responsible consumption tackles environmental, ethical, and social factors that entail economic effects. This began as green consumption, which considered environmental matters. After adding ethical matters, it came to be ethical consumption, and upon considering social matters, it became socially responsible consumption [1]. The consumption of organic food products can be considered as socially responsible, since it entails benefits for the environment, consumers, and smallholder farmers. This reverts the deterioration of the environment because organic food products are grown free from chemical pesticides and fertilisers [2,3]. For the consumers, it affords security regarding their health. Meanwhile, for the smallholder farmers, it provides economic gains [3], income to feed their families, and motivation to revive their ancestral practices. However, the consumer market in Mexico is poor—15% of organic production supplies the internal market, and 85% is exported to the United States, Germany, France, the United Kingdom, Canada, Italy, Switzerland, and Japan [4]. This creates a gap in the knowledge of consumer behaviour regarding organic food products in Mexico to identify a potential market for the purchase of these products and increase internal consumption.

In Mexico, organic food production is sustained by organised smallholder farmers of indigenous origin [5]. This is concentrated mainly in the states of Chiapas and Oaxaca, both with more than 70% of their population living in poverty [6]. To promote organic products in Mexico, the “Red Mexicana de Tianguis (traditional street markets in Mexico) y Mercados Orgánicos” (Mexican Network of Tianguis and Organic Markets) supports the commercialisation of these products [7]. A purchase intention model for organic food products can support smallholder farmers in redirecting their marketing strategies to attend to the segment of consumers of organic food products and, as such, increase their sales revenue. Armed with the knowledge of the factors involved, the markets can be efficient, and the producers can obtain a greater value for their products [8]; moreover, the consumers get good value, and the environment benefits from this cocreation of value.

The analysis of the consumption of organic products has been studied based on the theory of planned behaviour (TPB) [9,10]. In this theory, it is claimed that the relationships between attitudes, subjective norms, and perceived behavioural control are what influence the purchase intention [11]. However, previous studies also demonstrate that attitude is the predictor with the biggest influence on purchase intention, more so than subjective norms and perceived behavioural control [12,13,14].

Under the prism of the TPB, there is sufficient empirical evidence to prove the significant and positive relationship between attitude and purchase intention in different contexts of socially responsible consumption. For example, to explain the purchase intention of organic products for personal care [12], the purchasing of green products [13], or the purchasing of insect-based food products [14]. These previous studies give statistical evidence that in the TPB, attitude is the predictor that contributes the most to purchase intention, above subjective norms and perceived behavioural control, and provides the evaluation of the purchase benefits, such as organic food products being healthier, more nutritious, and more sustainable [15].

However, through the TPB, the motives that induce behavioural intention are not explained. The model of goal-directed behaviour (MGB) expands the TPB to include desire as the element that supplies the motives that cause an individual to act. The desire to achieve a goal is that which induces the behavioural intention bound to that goal [16]. For example, if an individual desires to achieve a goal such as caring for their health, and the purchase of organic food products helps them in achieving this goal, then the desire to care for their health will influence the individual’s purchase intention for organic food products.

The MGB model adds the motivational process to the theory of planned behaviour by adding desire. This model indicates that attitudes, subjective norms, and perceived behavioural control through desire are transformed into a behavioural motivation, with desire acting as a mediator of these relationships [16]. Desire draws on the passions and emotions of the individual to reach a personal goal [17], whether it be physical or related to health or appearance. However, desire also influences the decision of an ethical activity [18], decisions on green purchases [19], and socially responsible consumption [20]. In this study, the TPB model was expanded using the MGB model to analyse the mediating effect of consumer desire for organic food products in emerging economies. In this context, purchasing behaviours regarding organic food products are low, and the consumer perception regarding this kind of purchase is closely related to the price, with the benefits provided to one’s health, the care for the environment, and the direct economic benefits for smallholder farmers considered as much less relevant factors.

This study seeks to analyse the mediating effect of desire on the relationship between attitude and purchase intention. The purpose is to contribute to the literature on socially and environmentally responsible and ethical consumers, through the understanding of the mediating effect of desire, added as an extension of the TPB. In doing so, this study also seeks to generate knowledge on desire, attitude, and consumer purchase intention of organic food products in emerging economies, where a great number of agricultural producers are small and use organic food production practices and ancestral sowing techniques. The results might serve marketing practitioners in designing sales strategies for the socially responsible consumers segment of the market, which can positively impact the economy of smallholder farmer families, the local and countrywide economy, and the revival of organic production, which has larger labour demands [5,21], favouring the environment and the socially responsible market.

The rest of the document is organised in the following format. First, the context of the investigation is detailed. Second, the theoretical construction and formulation of the hypotheses are presented. Third, the method of investigation is described. Then, the results are exhibited. In the final sections, the discussion and implication of the study are presented, as well as the limitations and future investigations. 

## 2. Context of the Study: Organic Production in Mexico

In 2018, Mexico was one of the five main countries with emerging economies that are rich in biodiversity and natural resources [22]. It is one of the main producers of organic foods. In 2018, Mexico occupied the 40th place in the world regarding the surface area of organic farming and the 20th place in terms of the number of producers [23]. In ten years, it has shown a growth of more than 500%, from 71,780.71 tons of organic production in 2009 to 375,329.70 tons in 2019 [24]. Organic agriculture generates multiple jobs due to the labour needs, as it demands 30% more per hectare compared to conventional agriculture [5]. Thus, organic agriculture is an important economic activity.

Organic production has an advantage in the exportation market. 80% of the organic production in Mexico was allocated to exportation in 2020 [25]. It is an attractive market thanks to the surcharge of such products at international level, with a notable demand for organic goods in the American, German, French, English, Italian, Swiss, and Japanese markets [4,26].

In Mexico, organic agriculture is closely related to smallholder farmers from rural areas with the highest index of gaps in education, marginalisation, and poverty, such as Chiapas and Oaxaca [5,6,21]. Michoacán, Chiapas, and Oaxaca cover an expanse of 31,571.37 hectares (Table 1), which represented 67.46% of the sowed surface area of Mexico in 2019 [27]. The three crops with the highest sowed surface area in Mexico in 2019 were coffee, with 23,034.90 ha, avocados, with 9599.80 ha, and mangoes, with 4786.41 ha [27].

## 3. Theoretical Background

### 3.1. The Model of Goal-Directed Behaviour

The model of goal-directed behaviour (MGB) expands the theory of planned behaviour (TPB) to explain behavioural intention by adding variables to the model, given that it considers that the predictors of the TPB provide the reasons to act, but not the explicit motives which induce the intention to act; mostly driven by desire [16]. The TPB puts forward that the relationship between attitude, planned behavioural control, and subjective norms, with purchase intention, provides the reasons to act, but it leaves to one side the motivational, emotional, and habitual processes of the individual in the decision-making [11,16].

The MGB takes into account the impacts described by the TPB between attitudes, subjective norms, and perceived behavioural control on behavioural intention (volitional and non-volitional processes), but introduces desire as a mediator of these relationships, like a motivational process that leads the individual to decide or define a behaviour which is biased towards achieving a goal. Moreover, it integrates the relationships between anticipated emotions and the frequency of past behaviours regarding purchase intention, like effective and habitual processes, respectively [16].

The MGB proposes a distinction between desire and intention. To have the intention to carry out a behaviour, like buying organic food products, it is necessary to give oneself the desire to achieve a goal, such as caring for one’s health, protecting the environment, or supporting the producers. Desire is the motivation for the achievement of a goal, whilst intention is associated with carrying out the determined behaviour [16].

Although the TPB contemplates subjective norms, the perceived behavioural control, and attitude as factors that influence the behavioural intention [11], previous research provides evidence that shows attitude as the strongest predictor of purchase intention [12,13,14]. The MGB does not contemplate this relationship, but it proposes that, through desire, attitude can influence the behavioural intention, since it evaluates the indirect effect of desire in the relationship between attitude and purchase intention [16]. Nevertheless, the background of the MGB gives the reasons for considering the relationship between attitude and intention. Desires can be appetitive or volitive: appetitive desires are directed to consumption and are not based on reasons such as attitudes, whereas volitive desires are based on reasons and imply a motivational commitment [28]. It has been widely proven that a volitional component is necessary to have a behavioural intention proposed in the TPB [11]. This study analyses the consumption of organic food products, so desire can be appetitive. Therefore, the relationship between attitude and intention is considered, since it provides the volitional process, which desire for itself cannot provide. For example, a food consumption study based on the MGB shows that attitude has an important role in predicting behavioural intentions [29].

Additionally, the MGB strengthens this relationship with the mediation of desire as a variable that intervenes to provide the motives that induce purchase intention [16]. Therefore, the relationship between attitude and intention of the TPB is considered to provide the volitional process and to expand the understanding of the role of desire in this relationship.

**Figure 1:**
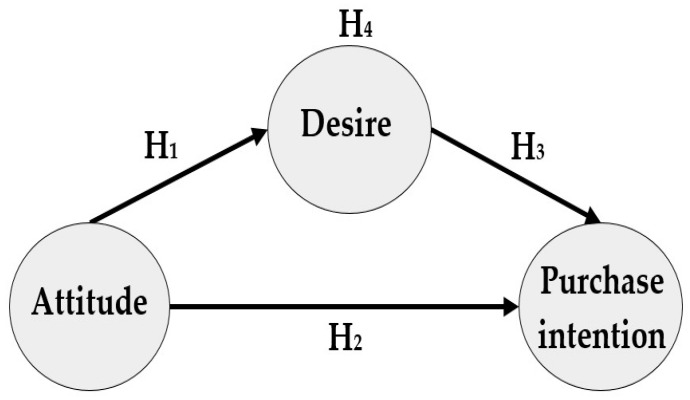
Hypothetical investigation model.

A study on food consumption based on the MGB finds that attitude influences desire, desire influences intention, and attitude influences intention, but does not prove whether desire has a mediating effect [29]. This study analyses the mediating effect of desire between attitude and purchase intention, and, for this, it evaluated the indirect effect of desire between the relationship of attitude and purchase intention, and the direct effect of the relationship between attitude and purchase intention, as described in Figure 1.

### 3.2. Attitude and Desire

In the literature on consumption, the discoveries regarding the influence of attitude on desire all correlate [29,30,31,32]. Attitude is measured as the grade of favourable individual perception towards a behaviour [11] and towards the desire, like a personal mental state in which a goal is reached or an action is carried out due to a motivation [16]. For example, in a food delivery study, it was shown that the favourable, good, and positive perception of drone food delivery services significantly influences the desire of the consumers to use this service when ordering food [29]. The consumer perception of visiting a food festival as desirable, enjoyable, attractive, exciting, and valuable influences the desire of consumers to visit an upcoming food festival [30]. A study on consumption in food trucks indicates that the evaluation of consumption in food trucks as advantageous, wise, pleasant, and attractive has a positive and significant influence on the desire to eat in these places when eating out [31]. Moreover, in wine tourism, the evaluation of participating in a wine tour as positive, valuable, beneficial, and necessary directly affects the desire to engage in such activity [32]. 

In other contexts of study, such as that of non-food consumption, evidence of this relationship was also found [33,34,35,36,37]. The consumption of sports goods has been found to have a positive and significant relationship between attitude and desire; the consumers’ perception of the purchase of sports items online as good, wise, worthy, or beneficial influences whether they want to purchase these items in the future [33,34]. Furthermore, in the study of consumers in airport shops, a positive relationship was found between attitude and desire; the consumers perceiving purchases in foreign airports as wise, pleasant, or enjoyable have a greater desire to buy in foreign airports on their next journey [35,36]. Likewise, consumers evaluating the use of a duty-free shop as good, beneficial, attractive, or wise exhibit a greater desire to purchase in these shops [37].

In responsible consumption, or responsible environmental actions, this relationship is also significant. When responsible consumption is analysed, a positive and significant relationship between attitude and desire is found [20,38,39,40]. For example, when airline customers have a favourable perception of voluntary carbon offsetting for society, this positively influences their desire to participate in this programme when purchasing their flights [38,40]. In addition, when consumers perceive staying in an environmentally responsible hotel as advantageous, wise, pleasant, and attractive, this influences their desire to stay in that kind of hotel when travelling [39]. Similarly, the favourable consumer perception of staying in eco-friendly hotels positively influences their desire to revisit them [20].

Additionally, when environmentally responsible tourist activities are examined, the positive and significant relationships between attitude and desire are also observed [41,42,43,44]. For example, when tourists favourably evaluate the mental, physical, environmental, and social benefits of cycling tourism, they increase their desire to partake in this activity [41]. Moreover, the favourable perception of slow tourism as positive, useful, valuable, beneficial, attractive, enjoyable, and necessary increases the desire of the tourist to participate in this kind of tourism [42]. The evaluation of tourists regarding travelling on an environmentally responsible cruise or visiting an environmentally responsible museum as good, wise, pleasant, beneficial, and attractive positively affects their desire to carry out the activity in the future [43,44]. This positive influence when the benefits of a specific behaviour are perceived, over the desire for it, could also be expected in the case of the consumption of organic food products. Thus, the following hypothesis is proposed:

**Hypothesis** **1.** *The attitude towards the purchase of organic food products positively and significantly influences the consumers’ desire to buy them.*

### 3.3. Attitude and Purchase Intention

In investigations of the purchase of sustainable products, attitude positively and significantly influences the purchase intention [12,13,45,46]. For example, when consumers perceive the purchase of organic personal care products as good or pleasant, it is likely that they will purchase these products [12]. Likewise, in the purchasing of green products, a positive and significant relationship is found between attitude and purchase intention, in that the favourable consumer perception regarding the purchase of green products increases the consideration and purchase expectancy for these products [13]. Furthermore, in studying the purchasing of organic coffee, it is found that attitude positively and significantly influences the purchase intention, and that a favourable perception towards the benefits of organic coffee, such as its quality and use, has an influence in making consumers try it, attempt to buy it, and suggest it to others [45]. Similarly, in a study of environmentally sustainable products, it was found that attitude positively and significantly influences their purchase intention, and that the consumer perception of the benefits of environmentally sustainable products on the environment, such as reducing both the waste of natural resources and contamination, as well as conserving the environment, increases their intention to use them, buy them if available, and actively look to acquire them [46].

In addition, when analysing other kinds of purchases, such as new purchases, it was found that attitude positively and significantly influences the purchase intention. The evaluation of the consumption of products made partly from insects as pleasant, relevant, tasty, and useful positively affects the intention to consume them and to try their consumption in the future [14]. The favourable consumer perception of the benefits of an environmentally responsible product positively influences the consumer in trying, considering, and recommending it. 

As the previously cited literature on consumption shows, attitude influences the purchase intention, which involves a volitional process. Although the MGB does not provide this relationship, it is important to consider this in consumer studies, since the appetitive desires are related to consumption and these do not involve a volitional process [28]. A study under the MGB found this relationship to be significant; in a food delivery study, the favourable perception of the use of drones for food delivery positively and significantly affects the consumer desire to use this service [29]. From here, the following hypothesis is proposed in relation to the influence of attitude over purchase intention in the case of organic food products:

**Hypothesis** **2.** *The attitude towards the purchase of organic food products positively and significantly influences their purchase intention.*

### 3.4. Desire and Purchase Intention

Previous research shows that consumer desire is a predictor of the intention of environmentally responsible behaviours, whether it be directed at a product or a service. For example, in the literature on environmentally responsible destinations, the influence of desire on the intention to visit an environmentally responsible museum is positive and significant. Thereby, a tourist with the desire to visit it also plans, is willing, and tries to do so [44].

Other studies on sustainable tourism also find a positive and significant relationship between desire and intention [20,39,41,42,43]. A tourist with the desire to be in an environmentally responsible hotel, to travel by bicycle, or to travel on an environmentally responsible cruise, plans and tries to stay in said hotel or go on this kind of trip [39,41,43]. Tourists’ desire to revisit an eco-friendly hotel positively influences them to have the intention to revisit it [20]. In turn, a tourist that has the desire for slow tourism is willing, has the intention, plans, will go out of their way, and will invest resources, to go on this kind of trip [42]. Desire acts as a motivational process and as the variable that, in sustainable behaviours, influences intention.

In addition, this relationship exists in other consumption behaviours. For example, investigations on purchases in airports show that desire is positively and significantly related to purchase intention [35,36,37]. Consumers with the desire to buy in an airport are willing to carry out their purchases during their next trip [35]. The desire to repurchase in an airport influences consumers to plan, have the intention, and strive to make repurchases in an airport [36]. In turn, when consumers have the desire to buy in a duty-free shop, they will plan, try to make their purchase in this kind of shop, and speak positively of it [37].

In the same way, studies on the purchases of sports goods find this positive and significant relationship, as consumers with the desire to buy sports items will plan and try to buy them [33,34]. As such, the following hypothesis is proposed for the consumption of organic food products:

**Hypothesis** **3.** *The consumers’ desire to buy organic food products positively and significantly influences their purchase intention.*

### 3.5. Attitude, Desire, and Purchase Intention

Desire can be understood as a positive interpersonal emotional state, capable of transforming a powerful motivation related to passions [17], which in turn wishes to achieve a goal to influence behavioural intention [16]. The model of behaviour, aimed at goals, suggests that desire mediates the relationship between attitude and purchase intention [16]. Upon connecting these variables, a stronger purchase intention is achieved. Consumers with goals tends to carry out actions which are motivated by their personal desire, based on a favourable perception towards the purchase of a good or service, which modifies their behaviour according to that which is perceived and desired. 

There is empirical evidence on sustainable behaviours and consumption, showing that attitude influences desire, which in turn influences intention [36,39]. For example, a consumer who is motivated by their personal desire to buy in airports based on the perceived benefits of this purchase tends to plan and make a stronger effort to make the purchase [36]. In sustainable behaviour, a tourist who is motivated by their desire to stay in an environmentally responsible hotel, and who perceives the benefits of this, will show a more solid plan, try, and exert effort to visit this kind of hotels [39].

Desire relates as much to passions as to ethical decisions [17,18]. Desire influences the decision of an ethical action when there is an ethical challenge [18], like in the decision of green purchases [19]. It can be understood that a consumer desire with ethical goals leads to an intention of socially responsible consumption such as the consumption of organic food products. The purchase motives for organic products have to do with caring for one’s health, the environment, and the producers [47]. So, even if little is known about the role of desire on the influence of attitude on purchase intention, it is expected that the desire to achieve an ethical goal such as caring for one’s health, protecting the environment, or supporting producers implies the performance of an ethical action, which may strengthen the positive influence of attitudes towards the consumption of organic food products on their purchase intention. As such, the following hypothesis is proposed:

**Hypothesis** **4.** *The consumers’ desire to buy organic food products mediates the relationship between their attitude towards the purchase of organic food products and their purchase intention.*

## 4. Method

A cross-sectional and explanatory study was done to prove the proposed hypotheses. To compile the data, a structured questionnaire was developed and given to consumers of organic food products from Mexico.

A pre-evaluation of the model was performed, which consisted of the specification of the structural model, the examination of the data, and an estimation of the model. A diagram was prepared which illustrates the hypotheses of the investigation (Figure 1). The variables were integrated starting with a reduction of factors through principal component analysis with Varimax rotation and Kaiser normalisation. The statistical package “Statistical Package of the Social Science (SPSS) version 20” was used for the reduction of factors. In addition, the content of the latent variables was examined and deemed to be reflective.

For contrasting the hypotheses, an analysis of the data was performed through Partial Least Squares Structural Equation Modelling (PLS-SEM). This technique was applied since it is recommended to use PLS-SEM for models with more than six indicators per construct [48,49], by means of PLS consistent data processing [50]. The evaluation of the results consisted of two phases—the assessment of the measurement model and the structural model [49]. Meanwhile, to evaluate the mediation effect, the test for the significance of the indirect effect and the test for the significance of the direct effect were also conducted [51,52]. The instrument was subjected to the evaluation of the results through the software Smart PLS version 3 with a sample of 204 respondents [53].

### 4.1. Questionnaire

A structured questionnaire was designed based on the literature review on the topic, to analyse the purchase intention for organic food products [9,10,16,32,54]. The final questionnaire consisted of 24 items measured with a 7-points Likert scale ranging from 1 (completely disagree) to 7 (completely agree). Four items measured the attitude, four measured the purchase intention, eight measured desire, and eight made up the descriptive data of the respondents. The used data collection method was the survey, and the information collection techniques were face-to-face interviews and self-administered interviews through forms implemented using social media.

### 4.2. Variable Operationalisation and Measurements

Attitude was defined as the favourable evaluation of a behaviour in relation to behavioural beliefs [13,36,37]. Its operational definition was the amount of consumer agreement with buying organic food products due to perceiving them as favourable products for their health and appearance, the environment, and society; it was measured using four items [9]. 

Purchase intention was defined as the disposition towards the purchase of organic food products [55]. Its operational definition was the amount of consumer disposition and loyalty towards buying organic food products; for its measurement, four items were used which included the loyalty of the customer, repurchasing, recommendation, and the search for the products [10,32,54]. The purchase intention variable was measured with a Likert scale of 7 points, from 1 (completely disagree) to 7 (completely agree).

Desire was defined as the mental state that leads an individual to have behavioural motives [40]. This variable was operationally defined as the amount of consumer encouragement to buy organic food products to achieve a personal goal regarding their appearance and health, the environment, and society. It was evaluated with 8 items, which were adapted to the context of the study with indicators on the environment, agricultural producers, and the appearance and health of the consumer [16]. This variable was measured with a Likert scale of 7 points, from 1 (very weak) to 7 (very strong).

The items used to measure the variables of the study are presented in Table 2. The operational variables have a common factor (reflective).

### 4.3. Sample

PLS-SEM can be applied with a small sample size, since it performs estimates of partial model structures and not of all the parameters at the same time; therefore, the minimum sample size requirement depends on the complexity of the individual equations [56]. To determine the minimum sample size for Partial Least Squares Structural Equation Modelling, the statistical power, level of significance, size effect, and number of predictors were specified [57,58]. A statistical power of 0.8 and a level of significance of 0.05 were specified, as they were considered acceptable for behavioural studies [59]. The size effect was considered average, as in similar studies [60]. Therefore, with a medium size effect, a statistical power of 0.8, a level of significance of 0.05, and two predictors according to the model, the minimum required size was 68 [57]. The size of the study sample covered the minimum requirement. 

Non-probability sampling was performed through quotas to fulfil the minimum required sample size for the PLS-SEM data processing. The sample selection was guided by two criteria: (1) consumers who have made a purchase of organic food products, (2) consumers who are older than 18 years of age.

The selection of sampling location was stratified by Mexican entities with a large demand for organic food products. The demand for organic food products was determined based on the application “Google Maps”, considering that the sites with the most offers for these products concentrated the consumers. In this application, the search was made in each Mexican entity with the key words: organic markets, organic tianguis, organic shops, with a result of 220 markets, tianguis, and shops specialising in the sale of organic products. Three Mexican entities with a large demand for organic products were chosen: Mexico City, with 20 establishments, the State of Mexico, with 16 establishments, and the state of Oaxaca, with 15 establishments.

The face-to-face interviews were given in five establishments selling organic food products, which were chosen randomly in the selected sampling location. The consumers who fit the selection criteria were invited to participate in the survey voluntarily. The rate of response was 1:2.

For the data collected via social media, the questionnaire was applied through Google Forms in social media groups on Facebook orientated towards the purchase of organic food products. The participants who were interested in responding to the questionnaire were selected through filter questions to make sure they complied with the two criteria of the selection sample. In each group, information on the investigation and the link to the questionnaire for its application were provided.

35% of the data was collected via social media groups on Facebook and 65% through face-to-face interviews. The 204 complete questionnaires were distributed between Mexico City (40.2%), the State of Mexico (30.9%), and the state of Oaxaca (28.9%). 37.7% of the consumers of organic food products were between the ages of 31 and 45 years; 36.3% between 18 and 30 years; and the rest, more than 45 years. 67.6% of the participants were women. 50.5% of the consumers had received tertiary education, and 23.6% had received postgraduate education. 43.1% of the consumers were single, 45.6% married, and 8.8% in a civil union. The descriptive data for the sample is shown in Table 3.

## 5. Results

### 5.1. Hypotheses Testing

The hypothesis tests were carried out through data analysis by PLS-SEM. Both the measurement model and the structural model were evaluated [49,51,52].

#### 5.1.1. Measurement Model Assessment Results

For the measurement model assessment, the consistent PLS algorithm was used [50]. The loadings for indicators, composite reliability, convergent validity, and discriminant validity were evaluated for the reflective measurement model [49]. In the evaluation of the loadings for indicators, the largest loadings stayed at 0.59, as, on occasion, smaller loadings than 0.7 contributed to the content validity [61]. The construct reliability was measured using Cronbach’s alpha coefficient, Dijkstra–Henseler’s rho (ρA), and composite reliability [62]. Reliability values of variables higher than 0.8 were considered viable for strict reliability [63]. For the convergent validity of the measurement model, a value equal or superior to 0.5 was considered from the average variance extracted (AVE) [48]. The discriminant validity was verified through the Fornell–Larcker criterion and cross-loading analysis. The square root of the AVE of a construct was verified to be greater than its correlation with other constructs following the Fornell–Larcker criterion [64]. Meanwhile, in the cross-loadings analysis, it was verified that no item would carry more intensity in other constructs than that which was measured [65]. The measure model assessment results are shown in Table 4.

#### 5.1.2. Structural Model Assessment Results

For the structural model assessment, the consistent PLS algorithm and Bootstrapping for consistent PLS were applied [50]. It consisted of evaluating the model’s collinearity, path coefficients, significance value, determination coefficients R^2^, the prediction value Q^2^, and the size of the effect f^2^ [66,67]. The values for the variance inflation factor (VIF), R^2^ and f^2^ were calculated using the algorithm for consistent PLS. The VIF values for the structural model were verified as being smaller than 5 to avoid multicollinearity problems [48]. The Q^2^ values were verified through Blindfolding with a value of D = 7 [68]. The path coefficients were calculated for the proof of hypothesis using Bootstrapping for consistent PLS with 5000 subsamples [49]. 

For the global model evaluation, the SRMR fit index (Standardised root mean square residual) was evaluated [66]. The mediation effect analysis, the direct and indirect effects, and their significance were calculated using Bootstrapping for consistent PLS with 5000 subsamples [49].

**Figure 2:**
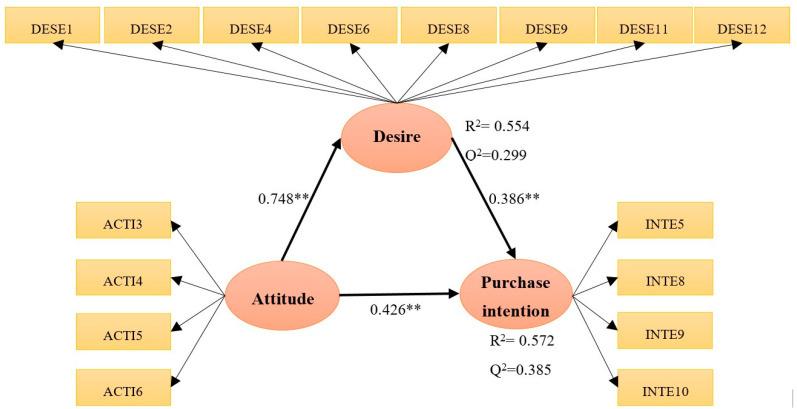
Determination coefficient and explanatory power of the hypothetical investigation model. ** *p* < 0.01.

The VIF, R^2^, f^2^ and Q^2^ values, the path coefficients, and their significance are shown in Table 5 and Figure 2. The Q^2^ value was 0.299 for consumer desire and 0.385 for purchase intention, which is to say, desire has a moderate amount of predictive relevance, and purchase intention has a strong predictive relevance [69]. The R^2^ values for consumer desire (R^2^ = 0.554), and intention (R^2^ = 0.572), are moderate [48]. To evaluate the global model, the SRMR fit index was 0.041. The model had a good fit for values under 0.08 SRMR [70].

In the case of Hypothesis 1, consumer attitude significantly influenced consumer desire (β = 0.748, *p* = 0.000). This is also the case for Hypothesis 2, where consumer attitude significantly influences purchase intention (β = 0.426, *p* = 0.001). Moreover, in the case of Hypothesis 3, consumer desire significantly influences purchase intention (β = 0.386, *p* = 0.001). For the evidence of Hypothesis 4, the mediation effect of consumer desire was evaluated.

The evaluation of the mediating effect of desire consisted of two parts: proof of the significance of the indirect effect and proof of the significance of the direct effect [51,52]. The results of the analysis of the mediation effect are shown in Table 6. Consumer desire had complementary mediation [71] for the relationship between consumer attitude and purchase intention.

## 6. Discussions and Implications

The data of this study confirm that consumer desire for organic food products has a mediating effect on the relationship between attitude and purchase intention of this kind of product. Consumer desire intervenes to increase the purchase intention for organic food products when consumers favourably evaluate the benefits for the environment, the consumer, and the producer when they consume this kind of product. 

In Mexico, studies on the consumption of organic products show that consumers are motivated by their health and the environment [72]. However, the results of this study go further and reveal that consumers are also motivated by caring for their appearance and for the economic wellbeing of producers. In this study, the consumers of organic food products demonstrate the main goals of their purchase intention. This indicates that the consumers of organic food products put their personal, social, and environmental desire first in their purchase intention; but also that the attitude towards the purchase plays a predictive role in desire, which contributes to perceiving that the benefits of consuming organic food products which will contribute to their goals are feasible. This confirms that desire can be bound together with ethical decisions [17]. Accordingly, in studies of socially responsible consumption, it is useful to include desire as a variable, as it positively influences the intention of socially responsible behaviour.

The discoveries of this study in Mexico contribute to the model of goal-directed behaviour to explain purchase behaviour. This contribution is based on the significant relationships found in the purchase intention model for organic food products and on its great explanatory power. Other similar studies also confirm the positive and significant relationships between attitude and purchase intention [45], between attitude and desire [38,39], and between desire and intention [20,73], although the main value of this study is to confirm the relationship among the three variables together, integrating desire as a mediator. Additionally, purchase intention has a larger determination coefficient than in studies where desire is not considered as a mediator [74]. It stands out that the addition of desire in the model has a significant role, given its complementary mediatory effect, and that it positively influences the relationship between attitude and purchase intention.

This study provides information to put forth marketing models directed towards socially responsible consumers. These marketing strategies can be focused on giving information to the consumer about the benefits of acquiring organic food products, not just for themselves, but also for the environment and the local producers. In Mexico, there is a closeness between the consumers of organic food products and smallholder farmers, given that the markets in which smallholder farmers operate are tianguis, fairs, and specialised shops where, most of the time, interaction is one-on-one. The interpersonal relationships that the consumer and the businessperson establish provide first-hand information to the consumers and increases their knowledge of the benefits of this kind of products and the implications of these purchases. However, the dynamic of local markets differs from other, more developed markets. For example, in big supermarkets where the consumer does not know the producer, nor is there one-on-one interaction, the consumer can only obtain information regarding the benefits of organic food products through secondary sources. This demands product strategies that provide more information on personal, environmental, and/or social benefits associated with the consumption of organic food products. This information will help future consumers who have the desire to care for their health, care for their appearance, protect the environment, and support the producers to know more about the benefits of purchasing organic food products, through marketing campaigns that will increase their purchase intention. Upon buying organic food products, future consumers would meet their goals and, as such, contribute to the economy of the agricultural producers of Mexico, as well as benefiting the environment.

## 7. Limitations and Future Research

One of the limitations of the study was that the analysis only included purchase intention and not purchase behaviour. The scope of the research limited the analysis of purchase intention, since it was not clear that the consumers, who had at any time purchased organic food products, had a clear purchase behaviour for this kind of products. As such, it is recommended that future investigations evaluate purchase behaviour. 

Another limitation of the study was the sampling location. This included local markets, specialised shops, fairs and tianguis. Supermarkets and other, more developed markets where the consumer does not have direct contact with the producers were not contemplated. It is recommended that future investigations include more developed markets as sampling locations to analyse sustainable behavioural intention.

In Mexico, the consumer obtains information on the benefits of organic food products through primary resources, given that, in most of the places where these products are bought, interaction is one-on-one (producer-consumer). However, in more developed markets, the consumers do not have this kind of interaction, and the information on organic food products is not obtained through primary sources. It is recommended that future investigations measure the effect of knowledge on the purchase intention of socially responsible consumers in more developed markets.

In accordance with the results, the majority of those interviewed were women and had a tertiary level education. As such, it is recommended that, in future research on socially responsible consumption, an analysis should be performed by population segments considering the gender and education level of the consumers and comparing them. Perhaps this phenomenon could lead to a generalisation with other market contexts.

It is finally recommended that the business strategies that incentivise the purchase of organic food products consider the social, environmental, and personal factors of the consumers. 

## Figures and Tables

**Table 1:** Entities with the largest surface area sowed with organic crops in Mexico in 2019.

Entity	Surface Area Sowed with Organic Crops (ha)
Michoacán	13,361.00
Chiapas	11,694.92
Oaxaca	6515.45
Nayarit	4770.80
Baja California	2484.00

Source: data taken from [27].

**Table 2:** Constructs and their indicators.

Construct	Indicator	
1. Attitude towards the purchase of organic food products	ACTI3	Buying an organic food product is good for the environment.
	ACTI4	Buying an organic food product is good for the smallholder farmers and their families.
	ACTI5	Buying an organic food product benefits my health.
	ACTI6	Buying an organic food product benefits my appearance.
2. Consumer desire	DESE1	My desire to buy organic food products to look good physically.
	DESE2	My desire to buy organic food products to look after my figure.
	DESE4	My desire to buy organic food products to look after my health.
	DESE6	My desire to buy organic food products to ensure my state of health.
	DESE8	My desire to buy organic food products to preserve the environment.
	DESE9	My desire to buy organic food products to be an environmentalist.
	DESE11	My desire to buy organic food products to contribute to the economy of smallholder farmers and their families.
	DESE12	My desire to buy organic food products to guarantee fair prices for smallholder farmers.
3. Purchase intention for organic food products	INTE5	I would search for places to buy organic food products.
	INTE8	I would recommend buying organic food products to my acquaintances.
	INTE9	I consider myself to be a loyal buyer of organic food products.
	INTE10	I intend to continue buying organic food products in the future.

Source: Adapted [9,10,16,32,54].

**Table 3:** Descriptive sample data.

Descriptive data	*n*	%
**Entity**		
Mexico City	82	40.2%
State of Mexico	63	30.9%
State of Oaxaca	59	28.9%
**Gender**		
Female	138	67.6%
Male	66	32.4%
**Age**		
18 to 30 years old	74	36.3%
31 to 45 years old	77	37.7%
More than 45 years old	53	26.0%
**Education**		
Primary	2	1.0%
Secondary	10	4.9%
Diploma	41	20.1%
Bachelors	103	50.5%
Masters	45	22.1%
Doctorate	3	1.5%
**Marital Status**		
Single	88	43.1%
Married	93	45.6%
Divorced	1	0.5%
Civil Union	18	8.8%
Widowed	2	1.0%
Separated	2	1.0%
**Level of income**		
Less than $9000	81	39.7%
$9000-$18,000	84	42.0%
$18,000-$36,000	26	13.0%
More than $36,000	13	6.4%
**Nationality**		
Mexican	201	49.8%
French	1	0.2%
Norwegian	1	0.2%
United States	1	0.2%
**Occupation**		
Student	30	14.7%
Employee	106	52.0%
Independent worker	44	21.6%
Homemaker	14	6.9%
Retired	6	2.9%
Other	4	2.0%

**Table 4:** Construct reliability, convergent validity, and discriminant validity.

Indicator	Load	AVE	ρA	CR	A	Fornell–Larcker Criterion			Cross Loadings		
						1	2	3	1	2	3
**1. Attitude towards the purchase of organic food products**											
ACTI3	0.953	0.791	0.943	0.938	0.937	0.889			0.953	0.700	0.694
ACTI4	0.903								0.903	0.687	0.631
ACTI5	0.915								0.915	0.667	0.671
ACTI6	0.776								0.776	0.595	0.537
**2. Consumer desire**											
DESE1	0.597	0.591	0.927	0.919	0.917	0.746	**0.769**		0.493	0.597	0.367
DESE2	0.647								0.518	0.647	0.415
DESE4	0.843								0.620	0.843	0.602
DESE6	0.835								0.633	0.835	0.576
DESE8	0.908								0.674	0.908	0.641
DESE9	0.723								0.530	0.723	0.519
DESE11	0.755								0.532	0.755	0.564
DESE12	0.792								0.566	0.792	0.583
**3. Purchase intention for organic food products**											
INTE5	0.908	0.711	0.917	0.907	0.903	0.715	0.702	0.843	0.664	0.621	0.908
INTE8	0.881								0.637	0.611	0.881
INTE9	0.672								0.453	0.505	0.672
INTE10	0.890								0.636	0.626	0.890

AVE- average variance extracted, ρA- Dijkstra–Henseler’s value, α- Cronbach’s alpha coefficient, CR- composite reliability. The square root of the AVE is on the diagonal (bold). Source: own elaboration with results obtained from the software Smart Partial Least Squares (PLS) version 3 [53].

**Table 5:** Structural model assessment.

Relationship	Β	*t*	*p*	f^2^	VIF
Attitude → Desire	0.748	14.142	0.000	1.254	1.000
Attitude → Purchase intention	0.426	3.415	0.001	0.195	2.254
Desire → Purchase intention	0.386	3.177	0.001	0.152	2.254

Β—path coefficient, *t*—*t* value, *p*—*p* value, f^2^—effect size, VIF- variance inflation factor. Source: own elaboration with results obtained from the software Smart PLS version 3 [53].

**Table 6:** Analysis of the mediation effect.

Relationship	Mediator	Direct Effect	Indirect Effect	Type of Mediation
Attitude → Purchase intention	Desire	0.426 **	0.289 **	Complementary

** *p* < 0.01. Source: own elaboration with results obtained from the software Smart PLS version 3 [53].

## Data Availability

The primary source data are not publicly available due to the institutional data policy. However, the data from this study are available upon request to the corresponding author.
